# Prediction of lymphovascular space invasion in patients with endometrial cancer

**DOI:** 10.7150/ijms.60718

**Published:** 2021-06-01

**Authors:** Sang Il Kim, Joo Hee Yoon, Sung Jong Lee, Min Jong Song, Jin Hwi Kim, Hae Nam Lee, Gyul Jung, Ji Geun Yoo

**Affiliations:** 1 Department of Obstetrics and Gynecology, St. Vincent's hospital, College of Medicine, The Catholic University of Korea, Seoul, Republic of Korea.; 2 Department of Obstetrics and Gynecology, Seoul St. Mary's hospital, College of Medicine, The Catholic University of Korea, Seoul, Republic of Korea.; 3 Department of Obstetrics and Gynecology, Yeouido St. Mary's hospital, College of Medicine, The Catholic University of Korea, Seoul, Republic of Korea.; 4 Department of Obstetrics and Gynecology, Uijeongbu St. Mary's hospital, College of Medicine, The Catholic University of Korea, Seoul, Republic of Korea.; 5 Department of Obstetrics and Gynecology, Buchen St. Mary's hospital, College of Medicine, The Catholic University of Korea, Seoul, Republic of Korea.; 6 Department of Obstetrics and Gynecology, Daejeon St. Mary's hospital, College of Medicine, The Catholic University of Korea, Seoul, Republic of Korea.

**Keywords:** endometrial cancer, lymphovascular space invasion, LVSI

## Abstract

**Objective:** Predict the presence of lymphovascular space invasion (LVSI), using uterine factors such as tumor diameter (TD), grade, and depth of myometrial invasion (MMI). Develop a predictive model that could serve as a marker of LVSI in women with endometrial cancer (EC).

**Methods:** Data from 888 patients with endometrioid EC who were treated between January 2009 and December 2018 were reviewed. The patients' data were retrieved from six institutions. We assessed the differences in the clinicopathological characteristics between patients with and without LVSI. We performed logistic regression analysis to determine which clinicopathological characteristics were the risk factors for positive LVSI status and to estimate the odds ratio (OR) for each covariate. Using the risk factors and OR identified through this process, we created a model that could predict LVSI and analyzed it further using receiver operating characteristic curve analysis.

**Results:** In multivariate logistic regression analysis, tumor size (P = 0.027), percentage of MMI (P < 0.001), and presence of cervical stromal invasion (P = 0.002) were identified as the risk factors for LVSI. Based on the results of multivariate logistic regression analysis, we developed a simplified LVSI prediction model for clinical use. We defined the “LVSI index” as “TD×%MMI×tumor grade×cervical stromal involvement.” The area under curve was 0.839 (95% CI= 0.809-0.869; sensitivity, 74.1%; specificity, 80.5%; negative predictive value, 47.3%; positive predictive value, 8.6%; P < 0.001), and the optimal cut-off value was 200.

**Conclusion:** Using the modified risk index of LVSI, it is possible to predict the presence of LVSI in women with endometrioid endometrial cancer. Our prediction model may be an appropriate tool for integration into the clinical decision-making process when assessed either preoperatively or intraoperatively.

## Introduction

Endometrial cancer (EC) is the most common cancer of the female reproductive tract in developed countries 1. According to the American Cancer Society, 66,570 new cases and 12,940 deaths are expected in the United States in 2021 2. In Korea, the incidence of endometrial cancer has been increasing, and it is now the most common gynecological cancer 3, 4.

Surgery is usually the primary treatment for EC, which consists of total hysterectomy and bilateral salpingo-oophorectomy with lymph node assessment 5. In the past, a full lymphadenectomy, including the pelvic and para-aortic nodes, was recommended in all patients with EC. However, many studies have reported that lymphadenectomy is associated with increased morbidity and no survival benefit in low-risk EC patients 6-8.

Thus, a more selective and tailored lymphadenectomy is recommended to prevent overtreatment.

Several risk-stratification models have been suggested to define low-risk EC: (1) Mayo and Mayo-modified classification; (2) European Society for Medical Oncology (ESMO) and ESMO-modified classification; and (3) Gynecologic Oncology Group-99 (GOG-99) classification 9-13. Korkmaz et al. compared these classifications to predict lymph node involvement in endometrioid EC clinically confined to the uterus. According to their study, the ESMO-modified classification seems to be the most accurate 14. The cornerstone of ESMO-modified classification is lymphovascular space invasion (LVSI) 12.

LVSI, the presence of cancer in lymphatic and/or vascular spaces within the uterine myometrium, is considered an essential prerequisite for lymphatic dissemination 15. Numerous studies have shown the strong correlation between LVSI, lymph node metastasis, and nodal recurrence 16-19. As LVSI is a poor prognostic factor in EC, it is important to determine the presence of LVSI during the decision-making process before performing a lymphadenectomy 20, 21. However, due to the inherent limitations of intraoperative frozen section (IFS) analysis, it is usually difficult to determine the presence of LVSI until the final pathology report is available 22, 23.

Previous studies have reported a positive correlation between the presence of LVSI and other risk factors, such as tumor diameter (TD), grade, and depth of myometrial invasion (MMI) 24-26. Based on this background information, we considered an indirect method to predict the presence of LVSI, using uterine factors such as TD, grade, and depth of MMI. The objective of this retrospective study was to develop a predictive model that could serve as a marker of LVSI in women with EC.

## Materials and Methods

This retrospective, multicenter study was performed with approval from the Institutional Review Board of the Catholic University of Korea (Approval No. XC21RIDI0051). The requirement for informed consent was waived owing to the retrospective nature of the study. The study was conducted in accordance with the principles of the Declaration of Helsinki.

This study included patients from six different hospitals, who were diagnosed with endometrial cancer and underwent primary surgical treatment, including total hysterectomy, between January 2009 and December 2018. Patients with non-endometrioid type of endometrial cancer, such as serous, clear cell, mixed type, and carcinosarcoma, were excluded. Other exclusion criteria were as follows: patients who did not undergo hysterectomy or received neoadjuvant treatment prior to hysterectomy.

Electronic medical records of the patients were reviewed to collect information such as age, body mass index (BMI), Eastern Cooperative Oncology Group (ECOG) performance status, medical comorbidities such as hypertension or diabetes, and date of the primary surgery. Pathology reports of the primary surgical treatment were reviewed for obtaining the International Federation of Gynecology and Obstetrics (FIGO) stage, FIGO grade, histologic type, TD, depth of MMI, LVSI, and lymph node metastasis. TD was defined as the largest diameter of the tumor. The percentage of myometrial invasion (%MMI) was defined as the deepest point of the myometrial invasion divided by the total uterine wall thickness in the same plane. Patients in whom the exact size of the tumor could not be determined from the pathology report were excluded. The exact size of the tumor could not be determined if the tumor exhibited a diffuse endometrial surface spread or if it was fragmented during the process of removal of the uterus by morcellation during laparoscopic surgery. Patients without an exact percentage of myometrium involvement were also excluded.

We assessed the differences in the clinicopathological characteristics between patients with and without LVSI. Fisher's exact test and chi-square test were used to compare the categorical variables. Continuous variables were compared using the Student's t-test or Mann-Whitney test. We performed univariate logistic regression analysis to determine which clinicopathological characteristics were the risk factors for positive LVSI status. Multivariate logistic regression analysis was subsequently used to estimate the odds ratio (OR) for each covariate. Variables with a P value less than 0.05 in the univariate analysis were included in the multivariate analysis.

Using the risk factors and ORs identified through this process, we created a model that could predict LVSI and analyzed it further using receiver operating characteristic (ROC) curve analysis. Statistical analysis was performed using R (version 4.0.3; R Foundation for Statistical Computing, Vienna, Austria).

## Results

### Baseline characteristics of the patients

A total of 1,169 patients underwent primary surgical treatment for endometrial cancer during the study period. Patients with non-endometrioid carcinoma (n=131), those who received neoadjuvant chemotherapy (n=6), and those with insufficient pathological information about the depth of MMI or LVSI (n=144) were excluded. Eight hundred eighty-eight patients were included in the final analysis. Among them, 201 (22.6%) were LVSI-positive and 687 (77.4%) were negative. The distribution of the variables between the two groups is shown in Table 1. LVSI positive patients were older (median 56.0 vs. 54.0 years, P=0.001), had lower BMI (23.8 vs. 24.9 kg/m^2^, P=0.004), more advanced FIGO stage (P < 0.001), higher histologic grade (P < 0.001), larger tumor size (4.0 vs. 2.2 cm, P < 0.001), deeper myometrial invasion (1.0 vs. 0.2 cm, P < 0.001), and more frequent cervical stromal invasion (23.9% vs. 3.6%, P < 0.001) than LVSI negative patients. There was no difference between the groups in terms of the ECOG performance status, medical comorbidities, and uterine wall thickness.

### Univariate and multivariate analysis

Univariate and multivariate analyses of clinicopathological variables are shown in Table 2. In univariate analysis, age > 60 years (P = 0.027), BMI (P = 0.007), tumor size (P < 0.001), histologic grade (P < 0.001), percentage of MMI (P < 0.001), and cervical stromal invasion (P < 0.001) were the risk factors for LVSI. In multivariate logistic regression analysis, tumor size (P = 0.027), percentage of MMI (P < 0.001), and presence of cervical stromal invasion (P = 0.002) were identified as the risk factors for LVSI. Regarding FIGO grades, grade 3 tumor was identified as an independent risk factor for LVSI (OR = 3.958, 95% confidence interval [CI] 2.250-6.961, P < 0.001), while grade 2 tumor showed marginally significant results (OR = 1.503, 95% CI 0.973-2.322, P = 0.066). The remaining two factors, age and BMI, were not significant. The model had an overall correct prediction rate of 82.4%, sensitivity of 78.1%, and specificity of 77.1% at the optimal cut-off value of 0.202. On ROC curve analysis, the area under the curve (AUC) was 0.842.

### Development of a prediction model for lymphovascular space invasion

**Figure 1:**
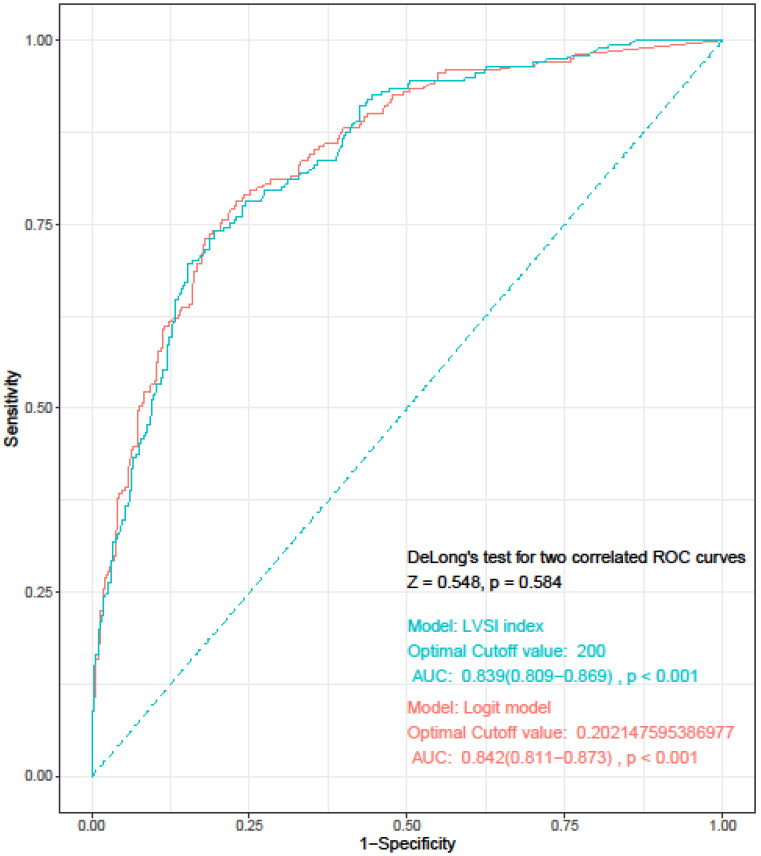
Receiver operating characteristics curve for “LVSI index” and logit model. LVSI: lymphovascular invasion; AUC: area under the curve.

Based on the results of multivariate logistic regression analysis, we developed a simplified LVSI prediction model for clinical use. We defined the “LVSI index” as “TD×%MMI×tumor grade×cervical stromal involvement.” TD (cm) and %MMI were calculated as absolute numbers. In case of tumors with only endometrial surface invasion without myometrial invasion, %MMI was scored as 1, and not as 0. We scored FIGO grade 1 as 1, grade 2 as 1.5, and grade 3 as 4. Absence of cervical stromal involvement was scored as 1 and presence as 2.5. Each score was weighted according to the ORs derived from the multivariate analysis. We calculated the LVSI for all patients in the study group and assessed the diagnostic accuracy using ROC curve analysis. The AUC was 0.839 (95% CI= 0.809-0.869; sensitivity, 74.1%; specificity, 80.5%; negative predictive value, 47.3%; positive predictive value, 8.6%; P < 0.001), and the optimal cut-off value was 200. DeLong's test was used to compare the LVSI and logit models, and showed that the two models were similar (P = 0.584) (Figure 1).

## Discussion

This study developed a risk assessment index to predict the probability of LVSI in women with endometrioid EC using uterine factors such as tumor diameter, tumor grade, and depth of myometrial invasion. The findings of this study indicate that it is possible to predict the presence of LVSI in patients with endometrioid EC.

**Figure 2:**
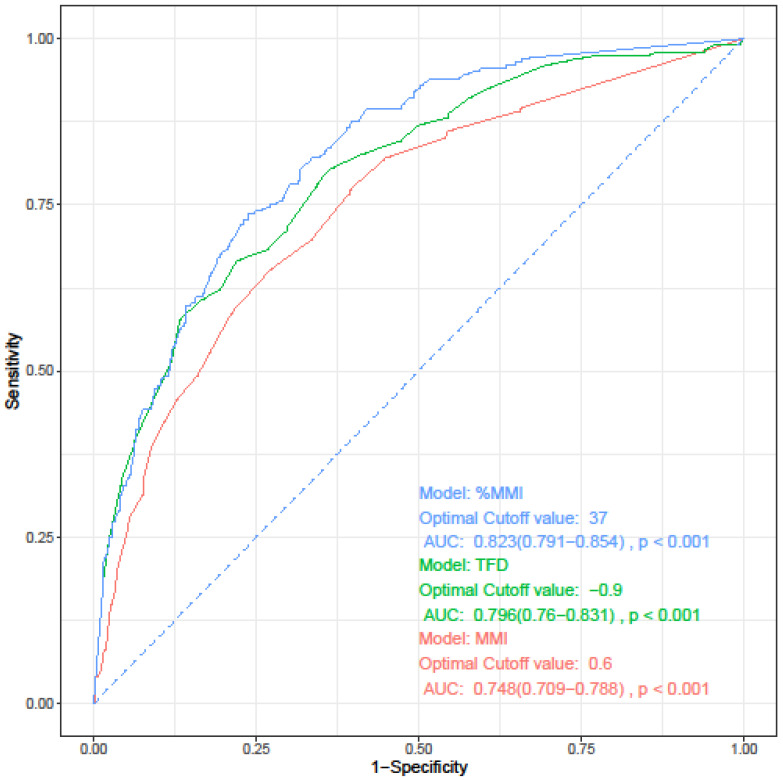
Receiver operating characteristics curve of each parameters for prediction of lymphovascular space invasion. AUC, area under the curve; CI, confidence interval; MMI, depth of myometrial invasion; TFD, tumor-free diameter; %MMI, percentage of myometrial invasion.

The depth of myometrial invasion is known to be related to both lymphovascular space involvement and prognosis. Three parameters have been proposed for measuring the myometrial invasion: depth of MMI, %MMI, and tumor free diameter (TFD). TFD refers to the distance from the deepest point of myometrial invasion to the uterine serosa 27, 28. Before developing a prediction model for LVSI, we investigated which of these three indicators best reflects LVSI. We performed univariate and multivariate logistic regression analyses, which were performed separately for each parameter while maintaining other variables as they were. All three parameters were confirmed as independent risk factors for LVSI (data not shown). On performing ROC curve analysis for the three parameters, %MMI was identified as the most useful parameter for predicting LVSI (Figure 2, Table 3).

Furthermore, LVSI has been proven to be a poor prognostic factor in previous studies 16-19. In addition, LVSI status is important in the decision-making process of lymphadenectomy. Numerous studies have reported that lymphadenectomy is associated with increased morbidity and no survival benefit in low-risk EC patients 6-8. However, lymphadenectomy is associated with improved overall survival in intermediate-and high-risk EC patients 29, 30. Patients with intermediate-and high-risk EC benefit from pelvic and para-aortic lymphadenectomy, and the survival benefit is superior to that of pelvic lymphadenectomy alone.

Thus, it is important to define low-, intermediate-, and high-risk EC. Moreover, LVSI status discriminates between low-risk and intermediate-risk EC. In stage IA (G1 and G2) with endometrioid histology, LVSI status is a determining factor for risk classification of EC. According to the ESMO-modified classification, without LVSI, it is classified as low risk, while with LVSI, it is classified as intermediate risk 12.

Although the LVSI status is an important risk marker and determinant in the decision-making process, clinicians usually do not know the LVSI status until the final pathology report is available. Some studies have reported the accuracy of LVSI by IFS analysis 22, 23, 31. Kumar et al. reported that a significant number of patients with low-risk endometrial cancer by IFS were upstaged and upgraded on final pathology. In addition, 31.7% of patients without LVSI on IFS analysis had a positive LVSI status on final pathology 22. Turan et al. reported that IFS only had a sensitivity of 50% for identifying LVSI 31. Another study by Pollom et al. reported that a major limitation of IFS analysis was the selective sampling of frozen sections. Because LVSI is often focal in nature, only a full review of accurate LVSI status 23. Despite the limitations of IFS analysis, prediction of LVSI status is crucial for the selective and tailored management of EC patients. Thus, we developed a prediction model for the LVSI status.

Meydanli et al. first suggested the “risk of LVSI index” by using well-known uterine factors such as the primary tumor diameter (PTD), percentage of myometrium involved, and the FIGO grade 32. They calculated the “risk of LVSI index” as “tumor grade × PTD × percentage of myometrium involved”, and scored grade 1 tumors as 1 and grade 2 or 3 tumors as 5. Additionally, tumors with a PTD < 30 mm were scored as 1, while tumors with a PTD ≥ 30 mm were scored as 3. The “percentage of myometrium involved” was represented with an absolute number. This index showed high accuracy in estimating the presence of LVSI at a cutoff point of 161.0 (sensitivity, 85.5%; specificity, 79.4%; AUC, 0.90; P < 0.001).

However, our LVSI index is different from that presented by Meydanli et al. First, we included the presence of cervical stromal invasion in the LVSI index as a risk factor. Second, we did not categorize the PTD but scored it as an absolute value. In our study group, the optimal cutoff point of PTD for predicting LVSI was identified as 2.7 cm on ROC curve analysis; however, categorization of patients with PTD of 2.7 cm was not significant in multivariate analysis (OR 1.466, 95% CI 0.933-2.302, P = 0.097). Third, we scored grade 1 tumors as 1, whereas grade 2 and grade 3 tumors were scored as 1.5 and 4, respectively, as described in the methods section. We adopted the “risk of LVSI index” suggested in a prior study into our study group and compared it with our LVSI index. ROC curve analysis and DeLong's test showed that our LVSI index had a higher accuracy than the “risk of LVSI index” for predicting the presence of LVSI (AUC 0.839 vs. 0.825, DeLong's test p = 0.022) (Supplementary Figure 1).

However, the accuracy of IFS analysis for TD, grade, and depth of MMI remains controversial. Karabagli et al. reported the reliability and accuracy of IFS analysis 33. The IFS results were in agreement with those of permanent section in 89.9 %, 88.6 %, and 100 % cases for grade, depth, and cervical invasion, respectively. On the other hand, in a study performed by Kumar et al., the IFS results were in disagreement with the permanent section results in 35% cases for the grade, 28% for the depth of myometrial invasion, and 13% for cervical involvement 22.

Factors responsible for inaccuracy of IFS analysis include erroneous interpretation, technical artifacts introduced by the frozen section technique, and inadequate sampling 34. Quinlivan et al. claimed that involvement of pathologists with specific expertise or interest in gynecological pathology in IFS analysis resulted in a higher accuracy rate. Evaluating multiple sections may also decrease the sampling errors 35. The accuracy of IFS analysis determines the real diagnostic performance of our model in the intraoperative setting.

Our study has several limitations. Firstly, the retrospective design may result in an inherent bias. Secondly, there was a lack of central pathology review. Finally, our findings depend on the final pathology results.

The strength of this study lies in the large number of patients with uniform endometrioid histology. As the clinical applicability of a prediction model is crucial, our model depends on well-known uterine factors obtained from the primary hysterectomy specimen, such as the tumor diameter, grade, and percentage of MMI, which can also be assessed by IFS analysis.

In conclusion, using the modified risk index of LVSI, it is possible to predict the presence of LVSI in women with endometrioid EC. Our prediction model may be an appropriate tool for integration into the clinical decision-making process when assessed either preoperatively or intraoperatively.

## Supplementary Material

Supplementary figure.

## Figures and Tables

**Table 1:** Baseline characteristics of the patients in the two study groups.

Characteristics	Total	(n=888)	LVSI+	(n=201)	LVSI-	(n=687)	P-value
Age, yr	54.0	(23-81)	56.0	(24-81)	54.0	(23-81)	**0.001**
BMI, kg/m^2^	24.7	(12.7-64.1)	23.8	(12.7-46.1)	24.9	(15.2-64.1)	**0.004**
Performance status							0.446
0-2	836	(94.1%)	187	(93.0%)	649	(94.5%)	
3-4	52	(5.9%)	14	(7.0%)	38	(5.5%)	
Medical comorbidities							
Hypertension	259	(29.2%)	68	(33.8%)	191	(27.8%)	0.098
Diabetes	120	(13.5%)	23	(11.4%)	97	(14.1%)	0.329
FIGO stage							**<0.001**
IA	587	(66.1%)	42	(20.9%)	545	(79.3%)	
IB	136	(15.3%)	44	(21.9%)	92	(13.4%)	
II	35	(3.9%)	13	(6.5%)	22	(3.2%)	
IIIA	27	(3.0%)	2	(1.0%)	25	(3.6%)	
IIIB	6	(0.7%)	1	(0.5%)	5	(0.7%)	
IIIC	72	(8.1%)	72	(35.8%)	0	(0.0%)	
IVA	3	(0.3%)	1	(0.5%)	2	(0.3%)	
IVB	22	(2.5%)	22	(10.9%)	0	(0.0%)	
Histologic grade							**<0.001**
1	478	(53.8%)	56	(27.9%)	422	(61.4%)	
2	305	(34.3%)	85	(42.3%)	220	(32.0%)	
3	105	(11.8%)	60	(29.9%)	45	(6.6%)	
Tumor size, cm	2.5	(0-16.0)	4.0	(0.5-16.0)	2.2	(0-12.0)	**<0.001**
Uterine wall thickness, cm	2.0	(0.4-9.0)	1.9	(0.4-6.0)	2.0	(0.5-9.0)	0.053
Invasion depth, cm	0.3	(0-5.5)	1.0	(0-4.0)	0.2	(0-5.5)	**<0.001**
Myometrial invasion, %	18.4	(0-100.0)	64.7	(0-100.0)	11.1	(0-100.0)	**<0.001**
Invasion ≥ 50% of the myometrium	246	(27.7%)	129	(64.2%)	117	(17.0%)	**<0.001**
Cervical stromal invasion	73	(8.2%)	48	(23.9%)	25	(3.6%)	**<0.001**

All values are expressed as the median (range) or number (%). Numbers marked in bold indicate p-values less than 0.05, which is considered statistically significant.* The time period from the date of surgery to the date of last adjuvant treatment administration. LVSI, lymphovascular space invasion; BMI, body mass index; FIGO, International Federation of Gynecology and Obstetrics.

**Table 2:** Univariate and multivariate analysis of factors associated with lymphovascular space invasion.

Characteristics	Univariate analysis			Multivariate analysis		
	OR	95% CI	*P*	OR	95% CI	*P*
Age, years						
<60	1 (Ref)	-	-	1 (Ref)	-	-
≥60	1.464	(1.045-2.051)	0.027	0.933	(0.615-1.416)	0.745
BMI, kg/m^2^	0.948	(0.912-0.986)	0.007	0.965	(0.921-1.011)	0.131
Performance status						
1-2	1 (Ref)	-	-			
3-4	1.216	(0.633-2.335)	0.558			
Hypertension						
No	1 (Ref)	-	-			
Yes	1.310	(0.934-1.836)	0.118			
Diabetes						
No	1 (Ref)	-	-			
Yes	0.786	(0.484-1.277)	0.331			
Tumor size, cm	1.489	(1.375-1.612)	<0.001	1.123	(1.013-1.246)	0.027
Histologic grade						
1	1 (Ref)	-	-	1 (Ref)	-	-
2	3.019	(2.068-4.406)	<0.001	1.503	(0.973-2.322)	0.066
3	10.543	(6.536-17.007)	<0.001	3.958	(2.250-6.961)	<0.001
Myometrial invasion, %	1.037	(1.031-1.043)	<0.001	1.026	(1.019-1.033)	<0.001
Cervical stromal invasion						
No	1 (Ref)	-	-	1 (Ref)	-	-
Yes	8.098	(4.833-13.567)	<0.001	2.623	(1.408-4.888)	0.002

Covariates with *P* < 0.05 on univariate analysis were included in multivariate model. OR, odds ratio; CI, confidence interval; BMI, body mass index; Ref, reference.

**Table 3:** Receiver operating characteristics curve analysis of each parameters for prediction of lymphovascular space invasion.

Parameter	Cut-point	AUC [95% CI]	Sensitivity	Specificity	P*	
					vs. MMI	vs. TFD
MMI	0.6	0.748 (0.709-0.788)	0.652	0.728	-	-
TFD	0.9	0.796 (0.760-0.831)	0.667	0.779	0.033	-
%MMI	37	0.823 (0.791-0.854)	0.736	0.761	<0.001	0.016

* The ROC curves were compared using DeLong's test. Values of cut-point of MMI and TFD is presented as centimeters, and %MMI as %. AUC, area under curve; CI, confidence interval; MMI, depth of myometrial invasion; TFD, tumor-free diameter; %MMI, percentage of myometrial invasion.
