# Supplementary material for: Prediction of lymphovascular space invasion in patients with endometrial cancer
Source: Int J Med Sci. 2021 Jun 1;18(13):2828–34. doi: 10.7150/ijms.60718 (PMC8241765; doi:10.7150/ijms.60718)
Supplement: Supplementary file 1 — Supplementary figure. [file ijmsv18p2828s1.pdf]

Supplementary Figure 1. Receiver operating characteristics curve for our “LVSI index” and the “risk of LVSI index” adopted to our study group

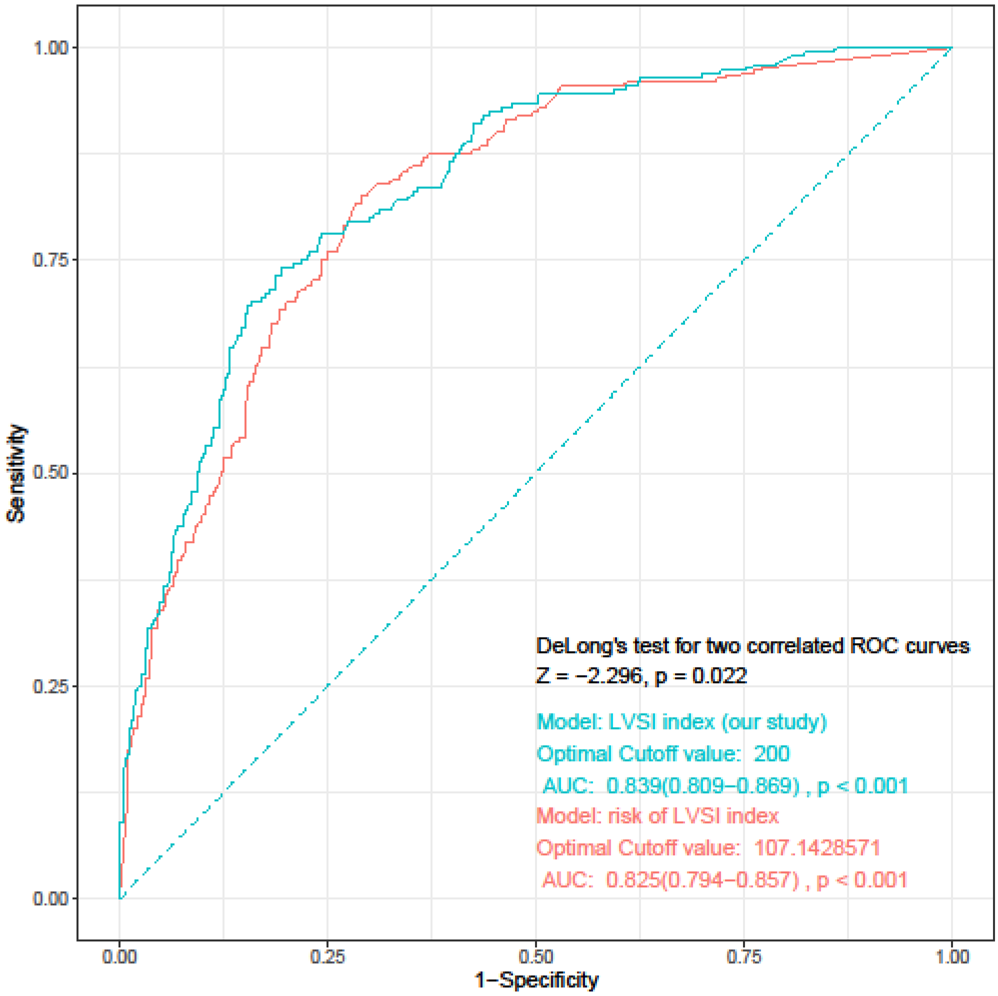

AUC, area under the curve.
